# Rural Household Preferences for Active Participation in “Payment for Ecosystem Service” Programs: A Case in the Miyun Reservoir Catchment, China

**DOI:** 10.1371/journal.pone.0169483

**Published:** 2017-01-03

**Authors:** Hao Li, Michael T. Bennett, Xuemei Jiang, Kebin Zhang, Xiaohui Yang

**Affiliations:** 1 School of Water and Soil Conservation, Beijing Forestry University, Beijing, China; 2 Beijing Forestry Society, Beijing, China; 3 College of Environmental Sciences and Engineering, Peking University, Beijing, China; 4 School of Economics and Management, Beijing Forestry University, Beijing, China; 5 Institute of Desertification Studies, Chinese Academy of Forestry, Beijing, China; Middlesex University, UNITED KINGDOM

## Abstract

Many payment for ecosystem services (PES) programs, such as the Slope Land Conversion Program (SLCP), are passive and require full participation by impacted households. In contrast, this study considers the alternative of “active and incomplete” participation in PES programs, in which participants are not obliged to contract their own land, and have the right to select into the program or not. This type of program has been popular over the last decade in China; however, there have been few studies on the characteristics of willingness to participate and implementation. As such, this paper uses the Choice Experiment (CE) method to explore ways for inducing effective program participation, by analyzing the effects of different regime attributes. The case study used to analyze participation utility was the Jing-Ji Afforestation Program for Ecological and Water Protection (JAPEWP), a typical active-participation forestry PES program, and a key source of water near Beijing in the Miyun Reservoir Catchment (MRC). Analyzing rural household survey data indicated that the program faces a variety of challenges, including long-term maintenance, implementation performance, cost-effectiveness, and monitoring approaches. There are also challenges with one-size-fits-all payment strategies, due to ineffective program participation or imperfect implementation regimes. In response, this study proposes several policies, including providing secure and complete land tenure to the participants, creating more local off-farm employment opportunities, designing performance-based monitoring systems that are integrated with financial incentives, applying differentiated payment strategies, providing capacity building to support forestation activities, and establishing a comprehensive implementation regime that would address these challenges. These policy conclusions provide valuable lessons for other active-participation PES programs as well.

## Introduction

"Payment for ecosystem services” (PES) is an effective policy mechanism that translates the external, nonmarket value of ecosystem services into individual economic incentives that motivate people to protect and restore ecosystems [1–6]. Over the last few decades, PES programs aimed at preventing environment degradation and alleviating poverty have developed at unprecedented rates in both developing and developed countries [7–12].

Willingness to participate is an inherent part of a PES program, and creates opportunities and the space for stakeholders to negotiate agendas, policies, programs, roles and partnerships [13–15]. Further, PES program participants can improve their livelihoods as a result of economic gain. This, in turn, can cause cascading socioeconomic and environmental effects [5, 16–18]. Engaging rural household participants, as key beneficiaries and leaders for implementation, is critical to PES program success. However, PES programs and projects often do not make strong claims about their participatory nature [19–20]. This leads to a paradox between more importance of and less attention on rural households’ willingness to participate in PES programs, and growing concern about it [21–22].

Internationally, many case studies have described rural household’s willingness to participate in PES programs [19, 23–31]. Some of these studies have empirically analyzed how incentive payments and benefits positively influence the decision to participate; decision criteria include contributions to household income and land opportunity costs [19, 26, 29, 32]. Some researchers have identified other key factors that induce willingness to participate in PES program, such as program flexibility and contract design [24, 30, 33], institutional system flexibility [19], greater land-tenure security [26, 28] and the coverage of agreement transaction costs [23, 24, 28, 30]. Other studies have focused on rural household demographics and farm-related features, such as human capital (age, education, skills, and ability to work) [26,31], economic income (on and off-farm income) [26] and farm-related features (land quality and size) [19, 26]. All of these also impact willingness to participate in PES schemes.

China leads global PES efforts [34]; most Chinese PES programs appear to be government-financed, top-down and campaign-style programs [35]. There are two types of typical PES programs in China from the perspective of local communities and rural household engagement. One type is called “full and passive participation." In these programs, participation is not entirely voluntary at the household level [15]. One example is the Sloping Land Conversion Program (SLCP, namely Grain for Green), one of the largest forestry PES and set-aside programs in the world [13, 36]. When cultivated lands are appropriated as project sites by the government, participants cannot decide when they can be released, and how the big land area is that can be enrolled into the program.

Over the last decade, many studies of SLCP have discussed the program’s impacts on rural households. These impacts include off-farm labor allocation [21, 37], grain supply [38], welfare income [3, 37], institution and market imperfections in program participation [15, 39–42] and the evaluation of program effectiveness and sustainability [13, 35–37, 41, 43–47].

The second type of PES in China is referred to as “incomplete and active participation.” With this type, rural households have the right to choose to participate or not participate in the programs. An example is the Sand Control Program in the Beijing-Tianjin Sand Source Regions [48–49]. More importantly, participants are not obliged to contract their own land with the programs. Instead, government-financed programs are designed to be implemented on state-owned or collective-owned marginal land, degraded land, or wasteland, where rural households have no or incomplete property rights [39, 41]. In fact, rural households mostly sign up to the programs in the form of short-term employment, rather than the previous format of a long-term contract.

As an international mega-city with a population exceeding 20 million, Beijing is suffering from an unprecedented water crisis [50–52]. Since 2008, a number of PES efforts have been led to address the crisis. These include projects led by the Beijing Municipal Government, such as the Paddy Land to Dry Land (PLDL) Program and the Jing (the abbreviation of Beijing)—Ji (the abbreviation of Hebei) Afforestation Program for Ecological and Water Protection (JAPEWP). These projects have been done around the Miyun Reservoir, the Beijing’s only surface water source for domestic water supply, and they convert land use systems to protect Beijing’s source water [53–55]. Among these programs, as a typical case of passive participation, the PLDL program received an evaluation of the welfare impacts on the rural households [5, 17]. However, little study has been done on the active participation program, JAPEWP.

This study uses the Choice Experiment (CE) method to explore ways to induce effective program participation, by analyzing the effects of various program attributes on participation utility. The goal was to address a variety of challenges, including long-term maintenance, implementation performance, cost-effectiveness and monitoring approaches. This research was needed due to ineffective program participation and imperfection implementation regimes in the JAPEWP program. This program is a typical active-participation type of forestry PES program, in the key source water area of Beijing, the Miyun Reservoir Catchment (MRC). The study provides valuable and unique policy insights into designing active and effective programs, as well as implementing efficient program regime policies for typical emerging active-participation PES programs.

## Materials and Methods

### Study area

The Miyun Reservoir, built in the 1960s, is in northeast Beijing, approximately 100 kilometers away from the city center. The reservoir originally had a surface area of 188 km^2^ and a maximum water storage capacity of 4.375 billion m^3^ and is the largest reservoir in the North China. It receives water mainly from the Chao and Bai Rivers [56–57]. The reservoir has generally supplied approximately 800 million m^3^ per year for urban water use; this accounts for 25% of total water used [58–59].

The MRC, primarily the upper watershed area, has significant strategic importance for the water security of Beijing [60]. However, an increasingly severe water crisis, caused by both water scarcity and pollution in the MRC, is threatening Beijing's prosperity and stability [50, 58, 61]. As an added factor, the total area of MRC is 15,788 km^2^, 80% of which is located in the separate Hebei Province. This cross-jurisdictional characteristic brings about a series of challenges in terms of development interests, for both upstream and downstream stakeholders [17].

Initiated in 2009, JAPEWP has invested a total of US$143.2 million to build an ecologically important 66,666 ha water protection forest within the Miyun and Guanting Reservoir Catchment of Hebei Province [62]. The program is designed to sustain and improve the forest’s water protection function, which depends on rehabilitating and re-establishing the forest ecosystem in key source water areas. However, this typical PES program of active household participation, like the Sand Control Program in Beijing-Tianjin Sand Source Regions, is relying on governmental support, more than local rural household’s willingness to participate.

First, young and capable labor forces are moving into urban areas to work as migrants [39, 46], leaving stay-at-home laborers, who are often older or infirm, to be involved in the program. For example, local governments, on behalf of the program, have hired laborers to plant and tend trees during implementation. However, due to their lack of physical strength and relevant knowledge, these stay-at-home laborers are unable to guarantee program quality and benefits in the long term, outside of completing on-site work. In addition, because there are only limited job opportunities provided in the program, only a small share of local households in the area benefit from these limited job opportunities. The majority of households are not able to enjoy the potential welfare benefits offered by the program. These conditions lead to PES program challenges that undermine sustainability, social inequity, and inefficiency, like other domestic and international PES program [30, 63–65].

This study involved a rural household survey in the MRC area of Hebei Province. Before that, however, the research team completed a pre-survey in Fengning, Luanping, and Chicheng counties in October 2012. Among these, Fengning county occupied more than 4,400 km^2^ of watershed area, covering two primary MRC water sources, the Chao river and Bai river [66]. As such, this county has significant importance in sustaining water supply to Beijing, and was selected as the study area.

Within Fengning county, based on pre-survey findings and reviews of local statistical yearbooks, Tanghe, Heishanzui, and Humaying Townships were selected as experimental sites. Of these, Tanghe Township is in the Bai river sub-catchment; Heishanzui and Humaying Township are located in the Chao river sub-catchments. In terms of local livelihoods, Tanghe Township is more of a traditional agriculture township compared to Heishanzui and Humaying Townships, where there are emerging mining industries (iron, gold etc.). As such, the rural households in Heishanzui and Humaying Townships have more diversified livelihood sources and depend less on agriculture than the Tanghe Township. Together, these three townships effectively represent MRC from the perspectives of geological scope and livelihood diversification.

### Methods

In our study, rural household’s willingness to participate (participation or no participation), which represents their preferences for attributes of the program, was treated as a discrete variable. This allowed the application of the CE method, using discrete econometric models to analyze the determinants of willingness to participate. CE is a Stated Preference Method rather than a Revealed Preference Method; as such, it relies on Lancastrian consumer theory [67] and random utility theory [68]. The goal is to place the decision-maker in a realistic frame of mind to compare a number of alternatives; each alternative is described in terms of some number of attributes [69–70].

Since the 1990s, CE has been applied to study public willingness to participate in agricultural and forest management [70–76], biodiversity conservation [77–78], waste management [79–81], and water-related policy [82–83]. In China, some case studies using the CE method have leveraged the SLCP assessment [15, 41, 42].

This case assumed that rural households maximize utility in a given choice set [69]. The choices applied in econometric models represent the individual’s utility [84]. Utility derived from any alternative depends on the alternative’s attributes, individual demographic characteristics, and alternative associated constants (ASC) [70, 85–86]. Thus, the probability of choosing the alternative with the highest utility is expressed as the participant's utility function with a random error term, ε [85].

Here, we assume that ε follows a Type I extreme value distribution (Gumbel distribution), with an independent and identical distribution across alternatives and individuals [68]. As such, the difference in error terms between different choices has a logistic distribution [87]. Therefore, the probability that the alternative with the highest utility is chosen can be estimated using a Conditional or Multi-nominal Logit (MNL) model. This model assumes that choices are consistent with the Independence from Irrelevant Alternatives (IIA) property [70]. Finally, the use of measurable attributes elicited from the CE survey at given level and range can estimate the unobservable total utility by estimating maximum likelihood [88–89].

Due to the non-linear nature of the MNL model, the raw coefficients do not reflect the effects of per unit attribute changes on the choice probability. Given this, the Marginal Effect (ME) is applied to measure the relative importance of each attribute affecting choice [41]. Based on ME, Marginal Willingness to Accept (MWTA), also known as the implicit price, provides an effective way to assess trade-offs among the different experimental attributes [85]. Because all choice attributes must have an associated price or monetary value [70], MWTA are point estimates of the value of a unit change associated with a non-monetary attribute [90]. MWTA provides a valuation of the significance that respondents put on a specific attribute of goods or service, and represents the Marginal Rate of Substitution (MRS) of money as compared to others attribute.

MWTA is estimated using the expression MWTA = -ME_n_/ME_p_, where ME_n_ and ME_p_ are the marginal effects of non-monetary and monetary attributes. Based on this expression, the total net willingness to accept (TWTA) at different utility levels can be estimated by: TWTA = -(V_0_-V_1_)/β_p._ In this expression, β_p_ is the estimation coefficient of price or monetary attribute. TWTA estimates the minimum bid that a rural household would be willing to accept to sign up for a program that yields utility V_1_, as compared to the value obtained from remaining with status quo V_0_ [41].

### CE design and data collection

During the October 2012 pre-survey, we developed a rough shortlist of choice attributes on the JAPEWP program policy. These were informed by consulting with focus groups, such as local authorities and rural households. A test using the draft choice sets consisting of these attributes was then tested in one village. After that, minor revisions were made to improve the efficiency of choice sets, in accordance with respondent feedback. Finally, the key factors assumed to most impact rural household’s willingness to participate in the JAPEWP program were listed into the final choice sets (Table 1). These included the attributes: the contract length (*CLENGTH*), freedom to leave (*RELEASE*), afforestation survival rate (*SRATE*), financial penalty (*PENALTY*), inspection method (*INSPECTION*), and cash subsidy (*CSUBSIDY*).

**Table 1:** Summary of the attributes in the CE design.

Attribute name	Attribute description	Level (coding)
*CLENGTH*	Contract length	1, 5, and 10 years
*RELEASE*	Freedom to leave at any time or not	1: can leave freely the program without penalty, 0: if otherwise
*SRATE*	Requirements for the plantation’s survival rate when inspected after implementation.	100%, 85%, and 75%^a^
*PENALTY*	Whether or not there are financial penalties for disqualification after inspection.	1: yes, 0: no
*INSPECTION*	The method of inspection.	1: irregular inspection; 0: otherwise
*CSUBSIDY*	Annual cash subsidy	750, 1500, 3000, 4500 and 7500 Yuan/ha/year^b^

^a^ According to the criteria issued by State Forestry Administration (SFA), the plantation’s survival rate must be no less than 85% in the first year and 80% in the third year.

^b^ 1Yuan = 0.13 US$

As Table 1 shows, attributes CLENGTH and SRATE have 3 possible levels; RELEASE, PENALTY and INSPECTION have 2 possible levels; and CSUBSIDY has 5 possible levels. Therefore, the design resulted in 60 = 3^2^×2^3^×5 total potential policy options. In our study, a choice set includes 4 options: alternative policies A, B, and C, and option D, which is no participation. These facilitate respondent choice behavior [69]. The experimental design has the objective of minimizing the number of combinations given to respondents, to enable statistical identification of the underlying preference functions [69]. To achieve this, policy options that were correlated with each other were eliminated, using the principles of CE orthogonal designs. These principles helped isolate the effects of individual attributes on choice [70]. This resulted in 24 retained policy options, along with the option of no participation. These were, in turn, grouped into 8 choice sets. Table 2 presents an example of a choice set.

**Table 2:** One example of choice set.

Attribute	Option A	Option B	Option C	Option D
*CLENGTH (year)*	10	5	1	No participation
*RELEASE*	Allow to release contract freely	Not allowed to release contract	Not allowed to release contract	
*SRATE (%)*	75	85	85	
*PENALTY*	No penalty	Penalty	No penalty	
*INSPECTION*	Irregular	Irregular	Regular	
*CSUBSIDY (Yuan/ha/year)*	750	3000	1500	

The field survey was jointly conducted by the Beijing Forestry Society (BFS) and Beijing Forestry University (BJFU) in August 2013. At the beginning of the field survey, with assistance from local township staffs, all villages within each selected township were grouped into rich, medium and poor groups according to their wealth status. Then, the survey team randomly sampled a number of villages from each group. Similarly, under the help of local village heads, a number of rural households were also selected with the above sampling approach in each sampling village (Table 3). The stratified sampling method ensured the sample representativeness with respect to wealth status and participant willingness, and minimized the sample bias to the largest extent possible.

**Table 3:** Counts and composition of sampled townships, villages, and households.

Sampled township	Count of sampled villages	Count of sampled households	Sub-catchment
Tanghe	4	96	Bai River
Heishanzui	5	101	Chao River
Humaying	4	102	Chao River
Total	13	299	

During the survey, the enumerators, led by local village heads, visited the sampled households and used the structural questionnaire interview to solicit respondent decisions on different choice sets. Improving on previous experience that the presence of village heads during the formal interview may impact respondent’s decisions and generate unnecessary choice bias. In order to ensure the validity and accuracy and the responses elicited, we thus requested that the village heads initially introduced the enumerators and respondents to know each other and would not be involved the following interview.

During the in-person interview phase, the enumerator first introduced the survey goals and background about the JAPEWP policy by reading through the following narrative wording on the first page of questionnaire for the respondent.

As the sole source of surface water, the Miyun Reservoir is important for supplying domestic water to Beijing city. The MRC area has a particularly strategic importance for providing water for the reservoir. The water resource status, however, is becoming increasingly impacted as the inflow from the MRC has decreased annually. Thus, the Beijing Municipal Government recently has made great PES efforts to restore the ecosystem services in the MRC area.

The JAPEWP program funded by the Beijing Municipal Government intends to restore ecosystem services in the MRC area and to guarantee the water security for Beijing via forestation approaches. The program participation is voluntary for the local rural household, that is, you can choose participate in or not participate in the program while there is no need to contract your land with the program like the SLCP program. However, once one decides to sign up to the program, the participant would be asked to obey the following program regulations. Firstly, the survival rate of the plantation should meet the qualification requirements proposed by the program. Secondly, the planting would receive inspection for the survival rate after afforestation, and might receive an economic penalty if the survival rate is insufficient. Finally, on the basis of a qualified performance the participant would receive some amount of cash subsidy each year. In addition, the program would not only have other requirements for the participant, such as contract length, release and penalty options but also provide relevant capacity building and technical services.

In order to assist the decision maker to formulate more efficient program policy, the organizer hopes to make sense of the real willingness to participate and the preferences to the implementation regime via the survey. Please make your choices and indicate the information related to you and your family following my instructions. Any of your responses and suggestions would be highly appreciated.

Then, the enumerators explained the meaning, level, and range of six attributes (Table 1). After a one-time tentative choosing process, in the formal experiment, the respondents were asked to select their preferred choices from 8 different choice sets, to elicit preferred program characteristics. In addition, enumerators requested data about respondent demographics, accurate as of the end of 2012. All 299 rural households selected participated in the interview, resulting in a 100% response rate. A total of 9568 observations were elicited from these sampled households.

## Results and Discussion

### Descriptive analysis

The socio-demographic characteristics of rural households affect choice utility (70, 89). As such, Table 4 reports the relevant variables surveyed.

**Table 4:** Summary of sampled household socio-demographic variables.

Variable name	Variable description	Mean	Standard deviation
*Demographic features*			
*HHSIZE*	Household size (persons per household)	4	1.4
*AGE*	Average age of per household member (years)	41	13.6
*EDUCATION*	Average years of education per household members (years)	6	2.3
*Socio-economic features*			
*FARMINC*	On-farm income (Yuan/person/year)	1917	1866.2
*OFFFARMINC*	Off-farm income (Yuan/person/year)	8258	10766.4
*FARMSUB*	Farm cash subsidy received (Yuan/person/year)	81	91.5
*FORSUB*	Forestry cash subsidy received (Yuan/person/year)	30	76.1
*Plot features*			
*FARMAREA*	Farmland area (ha/person)	0.1	0.1
*FORAREA*	Forestland area (ha/person)	0.3	1.0
*FARMNR*	Farmland plots per person	1.3	0.9
*FORNR*	Forestland plots per person	0.3	0.4

**Fig 1:**
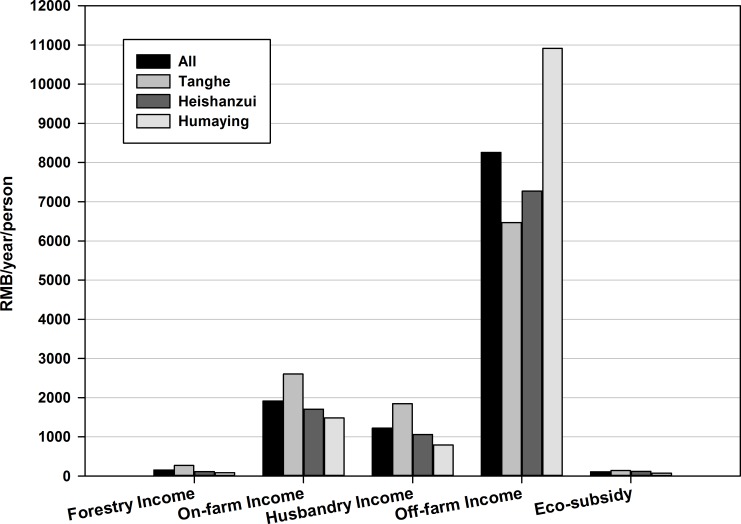
Comparison of income structure between townships in 2012 (Yuan/person/year). (1Yuan = 0.13 US$).

Fig 1 compares the income structure between townships.

Fig 1 shows that, based on higher on-farm, forestry, husbandry and eco-subsidy (i.e. SLCP and PLDL) incomes, the Tanghe township relies on traditional agriculture more than the other two towns. Tanghe township also appears to have richer land resources (0.6 ha/person), compared to Heishanzui (0.2 ha/person) and Humaying township (0.4 ha/person). Household size is almost equal between townships (4 persons/household).

In contrast, the higher off-farm income in the Humaying township suggests that local mining resource development may yield a more developed off-farm employment market. This form of well-established off-farm employment market could help households reallocate labor forces, and avoid over-supplying labor for traditional agriculture [39]. In summary, more traditional agriculture or eco-subsidy incomes rely more heavily on land resources. A more advanced off-farm economy, derived from higher off-farm income, may more effectively absorb labor resources from traditional agriculture.

### MNL modeling analysis

The MNL model was applied to analyze the effects of each attribute on choice utility. Table 5 presents model estimation results.

**Table 5:** MNL estimations of participant choices in the JAPEWP program.

Attribute name	Dependent variable: participation choices			
	All	Tanghe	Heishanzui	Humaying
*ASC*	-0.938***	-1.010***	-0.924***	-0.886***
	(0.045)	(0.080)	(0.077)	(0.076)
*CLENGTH*	-0.004	-0.033**	0.001	0.019
	(0.008)	(0.014)	(0.014)	(0.014)
*RELEASE*	0.812***	0.764***	0.702***	0.974***
	(0.063)	(0.109)	(0.108)	(0.110)
*SRATE*	-0.998***	-0.632***	-1.007***	-1.357***
	(0.113)	(0.194)	(0.193)	(0.200)
*PENALTY*	-0.869***	-0.874***	-0.898***	-0.842***
	(0.064)	(0.111)	(0.110)	(0.111)
*INSPECTION*	-0.060	-0.066	-0.106	-0.006
	(0.058)	(0.101)	(0.100)	(0.100)
*CSUBSIDY*	0.0002***	0.0002***	0.0002***	0.0002***
	(0.000)	(0.000)	(0.000)	(0.000)
Observations	9568	3072	3232	3264
LR chi2(6)	607.68	195.19	203.37	224.68
Prob > chi2	0.000	0.000	0.000	0.000

Notes: Standard errors are in parentheses.

*** Significance at 1%;

** significance at 5%

Overall, the outcomes of the Chi-square test indicate that the estimated results fit the empirical model well. Most estimated coefficients are statistically significant at the 1% level, except for the variables *CLENGTH* and *INSPECTION*. Increasing increments on both *RELEASE* and *CSUBSIDY* variable increase the associated utility level provided by the choice. The presence of either *SRATE* or *PENALTY* attribute decreases the utility. Every township showed similar estimation outcomes.

#### ASC

In this study, ASC reflects the utility derived from choosing to participate in the program, keeping all other attributes at status quo levels. In fact, the participant’s willingness to participate might be an interaction between local welfare status and the program attributes [41]. The significant negative correlation indicates that neither local welfare status nor maintaining the status quo or omitting the attributes can effectively induce active program participation. Therefore, in order to incentivize local willingness to participate as much as possible, policy measures are needed to adjust relevant attribute levels while improving social well-being via program implementation.

#### RELEASE & CSUBSIDY

Both *RELEASE* and *CSUBSIDY* attributes are significantly positively correlated with participation utility. The highest incremental utility occurs when participants are free to leave the contract. In other words, the ability for a participant to be freely released from the JAPEWP program contract has the largest positive effect on the willingness to participate.

On one hand, because the subsidy level is relatively low compared to the other income sources (Fig 1), the subsidy fails to induce active willingness to participate in the long term. On the other hand, without the freedom to leave the contract, rural households may worry about the risks and lost opportunities resulting from program participation. As a result, they are more likely to pursue job opportunities with the program that do not have a fixed term. How participants prioritize the ability to be released from the contract may threaten the program’s long-term sustainability of the program.

There is a big difference between active and passive SLCP participation; participating farmers are unlikely to exit as long as the payments are being delivered [43]. This may be because the majority of SLCP studies have been conducted in the remote areas of northwest and southwest China, where rural household willingness to accept subsidies is lower than around Beijing [15, 35, 39, 43]. More importantly, due to the appropriation of previously cultivated land, the SLCP can provide additional income sources, such as commercial forestry, to incentivize willingness to participate [41]. Therefore, increasing participating household income by diversifying income sources may be an effective way to induce more active willingness to participate in the JAPEWP program.

#### SRATE & PENALTY

As anticipated, the *SRATE* and *PENALTY* attributes negatively impact participation utility. A higher survival rate entails more labor input, as forestation is a labor intensive work in general among which planting, tending and fire patrolling all cost significant labor. Meanwhile, the relevant forestry knowledge and experience of the worker would be another pre-requisite because a skilled forestry laborer can provide better management [46]. As noted previously, the negative impact of *SRATE* reflects a lack of a sufficient and qualified local labor force due to higher opportunity costs. In addition, the stay-at-home laborers, who lack physical strength and relevant forestry knowledge, may worry about the possible economic losses that would occur if they experience a disqualifying survival rate. That is why the *PENALTY* attribute also has a negative effect on the rural household’s willingness to participate. Therefore, along with increasing and diversifying income sources, more local off-farm employment opportunities should be created to attract more qualified labor forces home [15, 37, 39, 41]. At the same time, technical training and service related to the program is needed to improve program implementation.

There are a few differences in the importance of specific variables between the townships. Tanghe Township households value *PENALTY* above *SRATE* (-0.874 VS. -0.632). As mentioned above, Tanghe Township depends more on traditional agriculture, whereas Humaying Township depends more on off-farm employment. Heishanzui Township falls somewhere in between. Households that are more vested and experienced in agriculture, with less exposure to off-farm labor markets, manage planted trees better [40]. As such, Tanghe Township households worry less about the survival rate requirement, as they are used to agricultural work and have richer forestry knowledge and experience. The situation in Humaying Township is the complete opposite. As such, different program conditions and mechanisms, consistent with regional socio-demographic characteristics, should be considered to improve the implementation regime.

#### CLENGTH & INSPECTION

Overall, neither the *CLENGTH* nor *INSPECTION* attributes significantly affect participation utility. Findings related to the contract length attribute were consistent with the “*RELEASE*” attribute discussed above. Because participants have stronger interests in having the freedom to be released from a contract, it is not unexpected for them to prefer a shorter-term contract, or to be insensitive to contract length at all. The insignificance of the *INSPECTION* attribute for all sample units indicates that ongoing inspection methods (regular or irregular inspection) do not provide sufficient guarantees to support program implementation. Therefore, more innovative policy methods and assessment mechanisms are needed.

To better understand the heterogeneity of household preference, we also assessed several other household-specific variables. These variables do not vary across household choices; as such, they drop out of the probability model. They can, however, be included in the model by assessing their interactions with the attributes that do vary across household choices [41]. Including these interaction terms in the model allows us to further examine the effects of household-specific characteristics on participation probability. Specifically, in addition to the household size, age and education years, the other variables, such as the household-specific land area, on and off-farm income and eco-subsidy, have been converted to dummy variables. These dummy variables at the high or low level are consistent with the first or third tertile that the sample belongs to. Table 6 presents these variables.

**Table 6:** Summary of household-specific variables,

Variable name	Variable description	Share of sample
**Household size**	Household size (persons per household)	-
**Age**	Average age of per household member (years)	-
**Education years**	Average education years of per household members (years)	-
**Low land area**	Bottom 33% of the sample in terms of land area per person.	36.79%
**High land area**	Top 33% of the sample in terms of land area per person.	33.11%
**Low on-farm income**	Bottom 33% of the sample in terms of share of household income comprised of on-farm income.	29.43%
**High on-farm income**	Top 33% of the sample in terms of share of household income comprised of on-farm income.	34.11%
**Low off-farm income**	Bottom 33% of the sample in terms of share of household income comprised of off-farm income.	31.77%
**High off-farm income**	Top 33% of the sample in terms of share of household income comprised of off-farm income.	33.78%
**Low eco-subsidy**	Bottom 33% of the sample in terms of share of household income comprised of eco-subsidy income.	32.78%
**High eco-subsidy**	Top 33% of the sample in terms of share of household income comprised of eco-subsidy income.	32.44%

Then, we added these household-specific variables interacted with each of the choice attributes into the MNL model estimation. Table 7 presents the regression results using interaction terms. There are a number of significant interactions between household-specific characteristics; all are consistent with expectations. In particular, low and high household on-farm income negatively affects willingness to participate. The effects of different income categories consistently imply the constraints on rural markets and institutions [15, 39]. The high on-farm income households who are limitedly exposed to market, such as off-farm job opportunities, land renting, have to rely more on traditional agriculture and cropping activities. The inefficient allocation of labor forces and materials to traditional agriculture might result in more worry about the risks and losses of opportunity costs due to program participation and would further inhibit their willingness to participate. In contrast, the low on-farm income households do not have a stronger willingness to participate, though they may have more off-farm income source. Program participation would allow the household to reallocate their labor forces; however, the constraints of exposure to market and the insecure forest land tenure are unable to ensure that they benefit sustainably from program participation [35, 39, 46]. Thus, the low on-farm income households would not like to take such higher opportunity costs. These findings are consistent with the discussion above.

**Table 7:** MNL estimation results using household-specific interaction terms associated with willingness to participate in the JAPEWP program.

Variables	CLENGTH	RELEASE	SRATE	PENALTY	INSPECTION	CSUBSIDY
***Interaction Term***						
**×**Household size	0.014***	-0.036	0.045	0.136***	0.025	0.00001**
	**(0.004)**	(0.039)	(0.032)	**(0.048)**	(0.038)	**(0.000)**
**×**Age	0.0003	-0.012***	-0.005	0.007	-0.006	-3.97e-07
	(0.000)	**(0.004)**	(0.004)	(0.005)	(0.004)	(0.000)
**×**Education years	0.002	-0.016	0.014	0.064***	0.030*	4.05e-06
	(0.002)	(0.018)	(0.015)	**(0.023)**	**(0.018)**	(0.000)
**×**Low land area	-0.024*	-0.091	-0.089	-0.111	-0.174	-0.00001
	**(0.013)**	(0.114)	(0.096)	(0.142)	(0.113)	(0.000)
**×**High land area	0.015	0.115	0.234***	0.147	0.085	0.00006***
	(0.012)	(0.109)	**(0.091)**	(0.135)	(0.107)	**(0.000)**
**×**Low on-farm income	-0.035***	-0.113	-0.301***	-0.327**	-0.302***	-0.00005***
	**(0.012)**	(0.106)	**(0.090)**	**(0.132)**	**(0.106)**	**(0.000)**
**×**High on-farm income	-0.025**	0.094	-0.228**	-0.519***	-0.134	-0.00007***
	**(0.012)**	(0.111)	**(0.093)**	**(0.140)**	(0.109)	**(0.000)**
**×**Low off-farm income	-0.011	-0.175	-0.035	0.082	0.003**	-5.54e-06
	(0.012)	(0.110)	(0.092)	(0.136)	**(0.109)**	(0.000)
**×**High off-farm income	0.009	0.116	0.101	-0.049	0.103	-6.36e-08
	(0.011)	(0.097)	(0.082)	(0.122)	(0.097)	(0.000)
**×**Low eco-subsidy	-0.011	0.067	-0.043	0.066	-0.042	-0.00003*
	(0.012)	(0.106)	(0.090)	(0.134)	(0.107)	**(0.000)**
**×**High eco-subsidy	-0.004	-0.185*	0.017	0.266***	-0.061	0.00002
	(0.012)	**(0.108)**	(0.089)	**(0.132)**	(0.105)	(0.000)
***Basic Attributes***									
ASC	-0.938***	-0.938***	-0.937***	-0.938***	-0.938***	-0.938***
	**(0.045)**	**(0.045)**	**(0.045)**	**(0.045)**	**(0.045)**	**(0.045)**
CLENGTH	-0.057	-0.004	-0.004	-0.004	-0.004	-0.005
	(0.039)	(0.008)	(0.008)	(0.008)	(0.008)	(0.008)
RELEASE	0.815***	1.584***	0.815***	0.815***	0.815***	0.810***
	**(0.063)**	**(0.342)**	**(0.063)**	**(0.063)**	**(0.063)**	**(0.063)**
SRATE	-0.994***	-0.997***	-0.961***	-1.006***	-1.002***	-0.990***
	**(0.113)**	**(0.113)**	**(0.306)**	**(0.113)**	**(0.113)**	**(0.113)**
PENALTY	-0.873***	-0.873***	-0.874***	-1.905***	-0.872***	-0.876***
	**(0.064)**	**(0.064)**	**(0.064)**	**(0.437)**	**(0.064)**	**(0.064)**
INSPECTION	-0.060	-0.058	-0.058	-0.059	0.074	-0.057
	(0.058)	(0.058)	(0.058)	(0.058)	(0.340)	(0.058)
CSUBSIDY	0.0002***	0.0002***	0.0002***	0.0002***	0.0002***	0.0002***
	**(0.000)**	**(0.000)**	**(0.000)**	**(0.000)**	**(0.000)**	**(0.000)**
LR chi2(17)	648.00	633.91	642.51	649.05	631.77	653.68
Prob > chi2	0.000	0.000	0.000	0.000	0.000	0.000

Notes: Standard errors are in parentheses.

*** Significance at 1%

** significance at 5%

* significance at 10%.

Therefore, the following potential measures should be taken into consideration to release the constraints on market and institution. Firstly, lowering dependence on traditional agriculture, while attracting more qualified labors to return home by creating new local income sources associated with JAPEWP program implementation, should be considered. Secondly, providing a set of complete and secure forest land tenure for the participants via forest land tenure reform would promote the willingness to participate and even further incent their land quality investment to some extent [39]. Lastly, as households may respond differently to conservation incentives [15], it is important to effectively target households that really need the program, consistent with their socio-demographic characteristics [43].

### ME estimation and simulation

The ME of the cash subsidy attribute suggests that increasing the cash subsidy by one Yuan increases the likelihood of program participation by 0.003% (Table 8). *Ceteris paribus*, an increase of 200 Yuan, results in a 0.5% increase in the likelihood of participation. These results suggest that depending on the importance of the cash subsidy is not a cost-effective way to motivate willingness to participate in the active type of program.

**Table 8:** Sampling household's MWTA for the choice attributes of the JAPEWP program.

Attribute name	All		Tanghe		Heishanzui		Humaying	
	ME	MWTA	ME	MWTA	ME	MWTA	ME	MWTA
***ASC***		4690.00		5050.00		4620.00		4430.00
***CLENGTH***	-0.001 (0.001)	21.32 (0.171)	-0.006** (0.002)	181.43 (0.209)	0.0002 (0.002)	-6.63 (0.178)	0.003 (0.002)	-101.52 (0.396)
***RELEASE***	0.143*** (0.011)	-4292.53 (3.429)	0.134*** (0.019)	-4205.62 (6.035)	0.123*** (0.019)	-3463.35 (4.040)	0.171*** (0.019)	-5268.65 (6.882)
***PENALTY***	-0.153*** (0.011)	4590.09 (3.416)	-0.154*** (0.019)	4806.79 (3.454)	-0.158*** (0.019)	4431.30 (4.484)	-0.147*** (0.019)	4553.06 (3.846)
***SRATE***	-0.176*** (0.020)	5274.15 (3.465)	-0.111*** (0.034)	3477.27 (3.275)	-0.177*** (0.034)	4970.04 (3.958)	-0.238*** (0.034)	7339.60 (7.402)
***CSUBSIDY***	0.00003*** (0.000)	-	0.00003*** (0.000)	-	0.00003*** (0.000)	-	0.00003*** (0.000)	-

Notes: The standard errors are in parentheses.

*** Significance at 1%

** significance at 5%

Turning to another continuous variable, a 1% increase in the required survival rate results in an 18% decline in the probability of participation. With the dummy variables, allowing participants the freedom to leave the program contract increases the likelihood of participation by 14%. As such, this variable should be considered an effective policy tool to induce willingness to participate. In contrast, the presence of the “financial penalty” attribute decreases the likelihood of participation by 15%.

The results associated with these attributes have important implications for policy designs, if the goal is to induce more household’s willingness to participate in the JAPEWP program. In addition, ME fluctuations among the three townships generally follow the above analysis of raw coefficients.

**Fig 2:**
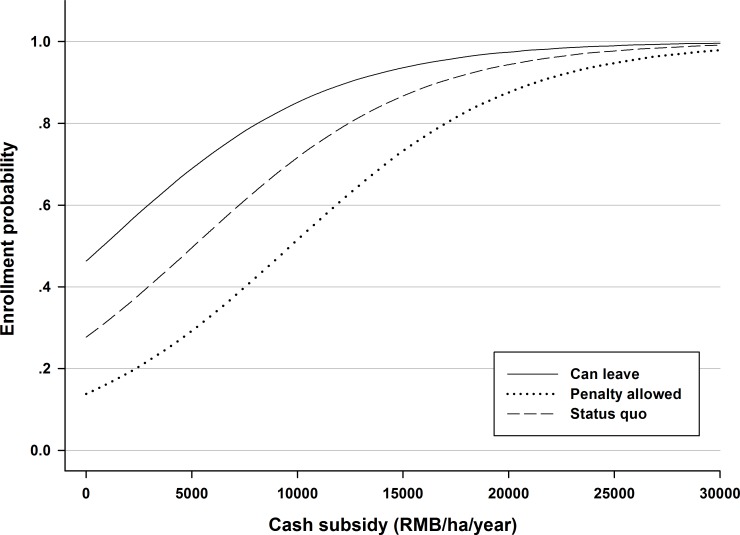
Simulation of enrollment probability for different choice attributes.

**Fig 3:**
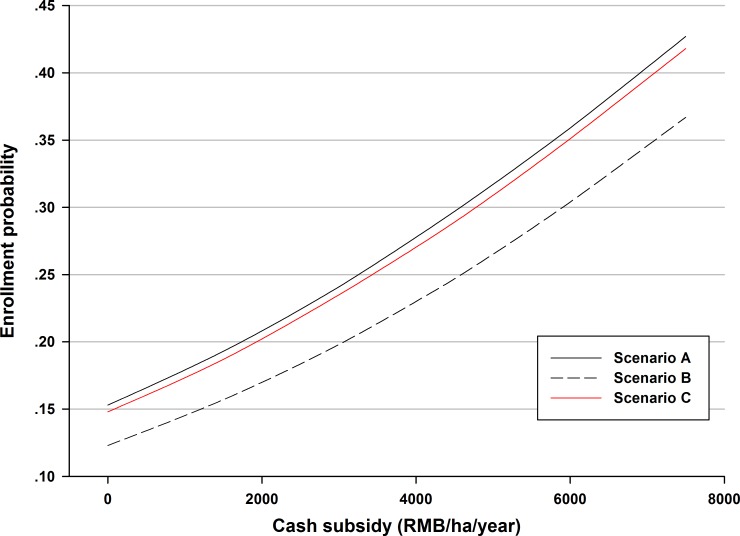
Simulation of enrollment probability for three policy scenarios.

Using the ME estimate outcomes, Figs 2 and 3 simulate the full probability distribution of program participation at different subsidy levels under different policy scenarios.

Fig 2 depicts the households' enrollment probability at different subsidy levels at the status quo level, with the “freedom to leave contract” attribute, and with the “financial penalty” attribute. As different policy interventions are introduced, the probability density curve shifts to the top or bottom. This type of policy tool can help decision-makers understand the effects of different policy interventions on enrollment probability and participation predictions at different subsidy levels.

For example, the probability density function shows that to achieve an enrollment probability of 0.5, a subsidy of no less than 5250 Yuan/year/ha should be paid to farmers, if all other attributes remain at status quo levels. However, once the “freedom to leave” and “financial penalty” interventions are introduced, the cash subsidy fluctuates to 750 and 9750 Yuan/ha/year, respectively. This indicates that different policy interventions could save significant cost for the program.

For the other two continuous variables, the “contract length” and “required survival rate” attributes, we designed three different policy scenarios to simulate enrollment probability; other attributes were kept at the status quo level. Scenario A required a 1-year contract and 75% survival rate; Scenario B assumed a 1-year contract, but required a 100% survival rate; Scenario C required a 10-year contract and 75% survival rate. Fig 3 suggests that the survival rate attribute impacts willingness to participate more than contract length. For example, when keeping the contract length steady at 1-year (Scenarios A and B) at a subsidy level of 4500 Yuan/ha/year, the enrollment probability is 0.297 and 0.247, respectively. However, when letting the survival rate equal 75%, *ceteris paribus* (Scenario A and C), the enrollment probability is 0.297 and 0.289, respectively. From this, it appears that technical training may help alleviate the pressure of survival rate.

### MWTA estimation

Table 8 reports the consumer surplus of different attributes derived from MWTA measures in Yuan/ha/year. A negative sign associated with an attribute assumes that program participants are willing to forego a certain amount of compensation surplus for an improvement in the attribute. Otherwise, the surplus compensation is not sufficient to promote the improvement, and there is a need for an additional subsidy [41]. When no policy interventions are introduced into the program, participants require an average minimum compensation (i.e. the value of ASC) of 4690 Yuan/ha/year, which is less than the ongoing PLDL program subsidy level (8250 Yuan/ha/year).

When considering specific attributes, the participants are willing to accept an income loss of 4293 Yuan/ha/year if they are free to leave the contract. The other three attributes are all associated with a participant need for additional compensation for each unit of improvement. Based on the equation introduced in the Methods section, if the program is implemented in a way in that adjusts the utility level from the status quo (V_0_) to the most desirable attribute level (V_1_) (i.e. 1-year contract, freedom to leave, 75% survival rate, and no financial penalty), the total net willingness to accept (TWTA) will drop to just 65 Yuan/ha/year. This further verifies the finding above that cost-saving goal can be achieved by reforms and additional supporting services.

When considering MWTA disparities between townships, the lowest MWTA value at the status quo level is for Humaying Township (i.e. the value of ASC). This suggests this township may have the greatest willingness to sign up for the program. Increasing local off-farm employment may increase participation rates at a given subsidy level [37, 39, 41]. More local off-farm employment opportunities may attract more qualified and capable labors to work locally, thereby allowing the township to provide sufficient labor resources to the JAPEWP program. Meanwhile, the sufficiency of local off-farm employment opportunities may make laborers less worried about potential losses in income or opportunities due to program participation. Tanghe Township is very different from Humaying Township, however. Therefore, differentiated interventions may be needed in different areas to achieve greater cost-effectiveness [15, 35].

## Conclusion and Policy Implication

Along with the dramatic development of PES programs overall in China, there are an increasing number of active-participation PES programs. In these programs, participants are not obligated to provide their own land to the program, and have the right to select into program participation or not. As such, it is projected that these programs can play more important roles in preventing environment degradation and alleviating poverty. However, with insufficient willingness to participate or ineffective implementation, this kind of program also faces challenges associated with long-term maintenance, implementation performance, and cost-effectiveness. Within this context, this study used the JAPEWP program as a case study to explore the characteristics of rural household preferences; the goal was to induce more active and effective program participation and establish an efficient implementation regime.

Program participation is currently voluntary at the household level. However, the program is unable to sustain long-term willingness to participate, due to the lower subsidy levels and higher labor opportunity costs. This study proposes that participant household income may be increased by diversifying income sources. In particular, the survey found that forestland tenure reform has not been completely undertaken in the area [42, 91–92]. Most wasteland or shrub land that has been or will be in the program, is currently under the control of the village collective, other than a small share of reserved and reclaimed plots. Land tenure imperfections undermine land quality investment incentives [39, 41]; as such, other participant income sources in addition to the cash subsidy (e.g. agro-forestry management) would have to be restricted. Hence, along with program implementation, secure and complete land tenure should be offered to the rural households as part of a complete and intensive forest land tenure reform.

Today, an increasing number of young and knowledgeable laborers have moved out of rural areas to work as migrants in cities. The survey also indicated that the majority of stay-at-home laborers are often old and ailing, and have less related knowledge and experience. The shortage of young and qualified laborers generates risks for program implementation and benefits (e.g. low survival rate). To encourage migrant labors to return home, more local off-farm employment opportunities would need to be created [39, 93–94]. For example, coupled with the municipal government’s scaling-up of agro-forestry management policies across Beijing [58], decision-makers should consider measures that introduce numerous cash crops (e.g. fruit trees, herb medicines) into the full MRC program, to release the constraint upon the local employment market [15, 39]. Meanwhile, different capacity building activities and technical services should be provided to the program participants; for example, instruction in seeding, planting, tending, and other farming activities.

The insignificant effect of the inspection method attribute on willingness to participate is in stark contrast to the significant impact of the financial penalty attribute. This suggests that the financial penalty is likely to ensure program implementation to some extent, but, overuse may decrease future rural household’s willingness to participate. This finding also indicates that the ongoing monitoring systems previously used in similar set-aside programs (e.g. SLCP) may not efficiently ensure program implementation [46]. A scientific performance-based monitoring system, that is integrated with and balances financial incentives and penalties, would benefit the program more.

Household willingness to participate in the program varies across townships with different socio-economic characteristics. However, programs still use a one-size-fits-all payment strategy, regardless of the interests of local rural households, and regardless of project location. Failing to spatially differentiate and target more effectively may result in losses in efficiency [8, 27, 43]. Hence, based on different levels of willingness to participate within a different area, program managers should consider specific conditionality, differentiation, and relevant mechanisms to increase the probability of program success [46]. For example, areas such as Humaying Township, where rural households have stronger interests in the program, should be provided more contract opportunities of longer durations, and a more rational subsidy level.

In conclusion, the JAPEWP program subsidy is no less than another similar PES program in the United States (e.g. Conservation Reserve Program, CRP), which had an average subsidy level of 705 Yuan/ha/year in 2016 [95]. As such, PES program success depends on a comprehensive implementation regime, rather than just the cash subsidy. The findings derived from simulating the enrollment probability confirm this. Applying a comprehensive policy portfolio can save significant amounts of subsidy costs [41]. Therefore, to motivate more active willingness to participate while saving program costs, a comprehensive implementation regime is needed. This regime should include many specific policy interventions, including pre-program consultations with rural households, alternative contractual arrangements, rational performance-based program monitoring, incentive mechanisms, and intensive capacity building services.

## Supporting Information

S1 File **Appendix A. Demographic data of rural household in the Miyun Reservoir Catchment area, China**.
(XLSX)

S2 File **Appendix B. Choice Experiment data and household-specific variables of rural household in the Miyun Reservoir Catchment area, China**.
(XLSX)
