# Correction: Rural Household Preferences for Active Participation in "Payment for Ecosystem Service" Programs: A Case in the Miyun Reservoir Catchment, China

**DOI:** 10.1371/journal.pone.0192546

**Published:** 2018-02-02

**Authors:** Hao Li, Michael T. Bennett, Xuemei Jiang, Kebin Zhang, Xiaohui Yang

There is an error in affiliation 1 for authors Hao Li and Kebin Zhang. Affiliation 1 should be:

School of Soil and Water Conservation, Beijing Forestry University, Beijing, China.
